# IBiSA_Tools: A Computational Toolkit for Ion-Binding State Analysis in Molecular Dynamics Trajectories of Ion Channels

**DOI:** 10.1371/journal.pone.0167524

**Published:** 2016-12-01

**Authors:** Kota Kasahara, Kengo Kinoshita

**Affiliations:** 1 College of Life Sciences, Ritsumeikan University, Kusatsu, Shiga, Japan; 2 Graduate School of Information Sciences, Tohoku University, Sendai, Miyagi, Japan; 3 Tohoku Medical Megabank Organization, Tohoku University, Sendai, Miyagi, Japan; 4 Institute of Development, Aging and Cancer, Tohoku University, Sendai, Miyagi, Japan; Wake Forest University, UNITED STATES

## Abstract

Ion conduction mechanisms of ion channels are a long-standing conundrum. Although the molecular dynamics (MD) method has been extensively used to simulate ion conduction dynamics at the atomic level, analysis and interpretation of MD results are not straightforward due to complexity of the dynamics. In our previous reports, we proposed an analytical method called *ion-binding state analysis* to scrutinize and summarize ion conduction mechanisms by taking advantage of a variety of analytical protocols, *e*.*g*., the complex network analysis, sequence alignment, and hierarchical clustering. This approach effectively revealed the ion conduction mechanisms and their dependence on the conditions, *i*.*e*., ion concentration and membrane voltage. Here, we present an easy-to-use computational toolkit for ion-binding state analysis, called IBiSA_tools. This toolkit consists of a C++ program and a series of Python and R scripts. From the trajectory file of MD simulations and a structure file, users can generate several images and statistics of ion conduction processes. A complex network named *ion-binding state graph* is generated in a standard graph format (graph modeling language; GML), which can be visualized by standard network analyzers such as Cytoscape. As a tutorial, a trajectory of a 50 ns MD simulation of the Kv1.2 channel is also distributed with the toolkit. Users can trace the entire process of ion-binding state analysis step by step. The novel method for analysis of ion conduction mechanisms of ion channels can be easily used by means of IBiSA_tools. This software is distributed under an open source license at the following URL: http://www.ritsumei.ac.jp/~ktkshr/ibisa_tools/

## Introduction

Ion channels are a major class of proteins and play essential roles in living systems [1]. Elucidating the ion conduction mechanisms of ion channels is of paramount importance not only for biology but also for medical sciences because dysfunction of a channel can cause several diseases [2]. In the past several decades, experimental efforts have illuminated the molecular mechanisms of ion channels, mainly by means of electrophysiology [3] and X-ray crystallography methods [4]. Although recent advances in experimental methods allow for successful dissection of many characteristics of ion conduction [5–7], examination of atomic details of ion conduction dynamics is not straightforward due to the complexity and microscopic scale of this phenomenon. In order to gain insights into this process, theoretical approaches, especially the molecular dynamics (MD) method, have been extensively applied, based on the wealth of the structural information obtained by crystallography [4,8–10]. Previously, it was difficult to simulate ion conduction because it is a relatively rare event relative to the time scale accessible to the MD method [11]. Nonetheless, recent continuous innovations in computer technologies are amplifying the potential of the MD method, and long-term simulations involving many ion conduction events can be performed [12–14].

On the other hand, a long-term MD simulation generates a huge amount of trajectory data, which consist of Cartesian coordinates of each atom in each snapshot. For example, in a 1 μs simulation of a system with 100,000 atoms, taking a snapshot every 1 ps produces more than 1 TB of trajectory data. Abundance of data inevitably makes it difficult to extract information and knowledge from the data, especially for complex phenomena such as ion conduction processes. For example, because K^+^ channels have a series of ion-binding sites and K^+^ ions permeate the pore in the single-file manner [3], characterizing motions of multiple ions at multiple ion-binding sites requires analyses of multidimensional space. Other research groups have utilized a two-dimensional (2D) map of the potential of the mean force, whose reaction coordinates are the position of the first ion and the midpoint between the second and third ions [12,15]. Although this method has successfully captured certain characteristics of ion conduction mechanisms, the projection of the multidimensional space onto 2D space masks details of the motions. Movements of the second and third ions are not always coupled, and sometimes, the number of ions in the pore can be two or four. Thus, an alternative approach to characterization of motions of ions in a channel pore is required.

In our previous reports, we have presented a new method for analyzing the ion conduction mechanisms of an ion channel using MD trajectories, called *ion-binding state analysis* [16,17]. This method captures the ion motions as a series of discretized ion-binding states, which are defined in terms of the existence of ions at each ion-binding site. The landscape of the potential of mean forces can be visualized as a network diagram named *ion-binding state graph*, and each ion conduction event can be expressed as a cyclic path in this network. We have previously applied this method to analyze 6.5 μs simulation data including more than a hundred ion conduction events and clarified preferences of two ion conduction mechanisms, *i*.*e*., the knock-on [3] and the association/dissociation (A/D) [18], with respect to the two conditions, *i*.*e*., the ion concentration and membrane voltage.

Here, we present a freely available, open-source software toolkit for ion-binding state analysis, called IBiSA_tools. A C++ program and a series of Python scripts process MD trajectory files with ion conduction dynamics and return diverse information about ion conduction events, such as ion distributions in a channel pore, the network diagram of ion-binding states as an energy landscape, and a classification of each ion conduction event. This toolkit may be useful for researchers who study the mechanisms of ion channels by the MD method.

This report is organized as follows: “Implementation” describes an overview of IBiSA_tools and functions of each component. “Results and Discussion” presents results of analyses for a sample trajectory, which is an all-atom MD simulation of a K^+^ channel. “Conclusions” discusses advantages and limitations of IBiSA_tools.

## Implementation

**Fig 1:**
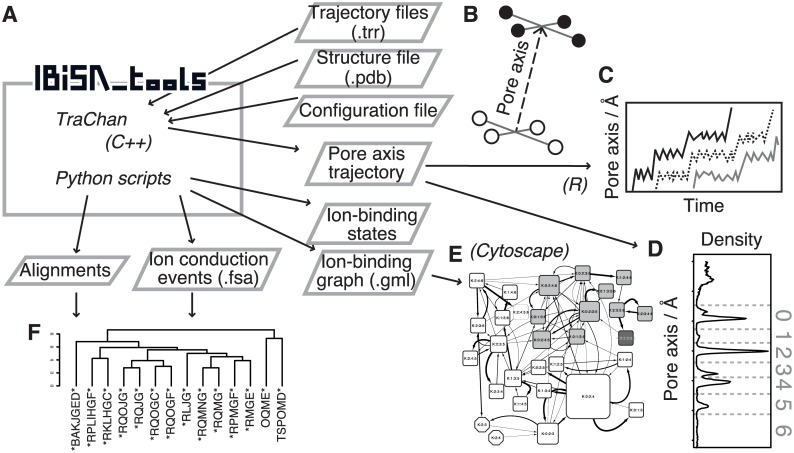
An overview of IBiSA_tools. (A) A summary of the components of IBiSA_tools (dashed rectangle) and their input and output. (B) A schematic image of a definition of the pore axis. Two sets of atoms (the open circles and filled circles) are specified by users. The pore axis is defined as the line from the center of the first set to the center of the second set. (C) An image of the generated figure depicting time courses of ions in coordinates. (D) An image of the generated figure of frequency of ions across the pore axis. (E) An image of the ion-binding state graph. The nodes indicate ion-binding states, and arrows represent the observed transitions between states. (F) An image of classification of ion conduction events. Each string below the dendrogram corresponds to each ion conduction event.

IBiSA_tools consists of a C++ program and several Python scripts (version 2.6 or later is required). For visualization of the results, some R scripts are also included. The overview of this toolkit is provided in Fig 1.

First, a series of MD trajectory files and an initial structure file are needed as input (*—fn-trr* setting in the configuration file). Because the current version accepts only .trr format (GROMACS), trajectory files written in other formats have to be converted into .trr format with some other tools, *e*.*g*., VMD [19] or MDAnalysis [20]. See the documentation for brief instructions on the file conversion. The C++ program named *TraChan* parses a trajectory and converts Cartesian coordinates of ions into the coordinates along the channel pore axis, which is defined by two sets of atoms as the bases; the line through the center of the first set to that of the other set is defined as the pore axis (Fig 1B; the settings—*pore-axis-basis-from* and—*pore-axis-basis-to* in the configuration file specify these sets of atoms). The output files record positions of ions along the pore axis and the radial distance from the pore axis. The former can be visualized with an attached R script (Fig 1C). The histogram of frequency of ion coordinates across the pore axis is analyzed by means of *ion_histogram*.*py* and an attached R script (Fig 1D). When the histogram shows a well-separated multimodal distribution like Fig 1D, boundaries of a series of ion-binding sites can be defined by the pore axis coordinates at the local minimum between every successive two peaks (the coordinates of boundaries are specified by the keyword—*site-boundary* in the configuration file). If the peaks cannot be separated by certain values on the pore axis, then the definition of the pore axis should be revised. Note that, there is no standard to determine whether a hypothetical peak is treated as separated two peaks or a broad single peak based on the density distribution. In such twilight cases, ion-binding sites can be defined in terms of physicochemical perspective. For example, in a K^+^ channel, each ion-binding sites is formed as an octahedral site by the backbone oxygen atoms of the SF. In addition, the boundary of ion-binding sites on the radial coordinate also can be defined (*—site-max-radius* setting in the configuration file). This setting is not important for usual cases because the channel pore is narrowed by the protein structure and the range of ion-binding sites on the radial coordinates is sterically defined by the protein structure. Details of the other settings are described in the software documentation. Next, program *site_occupancy*.*py* analyzes what ion-binding sites have ions in each snapshot. We define the ion-binding state of each snapshot as a set of ion-binding sites with ions. For example, when a series of ion-binding sites are named as 0-origin numbers from the extracellular side to the intracellular side (Fig 1D) and K^+^ ions bind to the ion-binding sites 0, 2, and 4 at the same time, the ion-binding state of this snapshot is referred to as K:0:2:4. A transition from K:0:2:4 to K:2:4 means that the outermost ion is released from the pore into the extracellular fluid. Program *analyze_ion_path*.*py* shows what ion-binding sites were passed by each ion during each conduction process.

To summarize ion-binding states observed in a trajectory, the network diagram called *ion-binding state graph* is generated (Fig 1E). A node represents an ion-binding state, and an edge between two nodes denotes transition between them. The probabilities of each node and edge are depicted as a size of the node and width of the edge, respectively. This graph can be drawn with standard network analysis tools, *e*.*g*., Cytoscape [21] via an output file generated by *analyze_site_state*.*py* written in the standard graph format Graph Modeling Language (GML). In the ion-binding state graph, a simulation trajectory is expressed as a single path, and the latter can be decomposed into a series of cyclic paths, each of which starts from the most stable state and reaches the same state. When a cyclic path includes both ion association and dissociation processes, the path corresponds to an ion conduction event. A list of cyclic paths is obtained by means of program *extract_cycles*.*py*.

To characterize patterns of ion conduction events, all the cyclic paths are compared and classified into some groups. Program *convert_state_characters*.*py* assigns a unique character to each ion-binding state. For example, the * character (asterisk) is assigned to the most stable ion-binding state, which is K:0:2:4. On the basis of this assignment, program *cycle_to_sequence*.*py* converts cyclic paths into sequences. The similarities between ion conduction events are assessed on the basis of the sequence alignment, which is performed by the *dp_align*.*py* program. This alignment task requires a matrix of similarities among ion-binding states (characters), which is generated by *make_score_matrix*.*py*. This program uses a simple similarity definition: the similarity score between the same states is 1.0, that between states with the same number of ions is 0.5, otherwise it is 0.0. Program *align_similarity*.*py* generates a similarity matrix of ion conduction events. This matrix can be easily analyzed by data analysis software; an attached R script performs hierarchical clustering and draws a dendrogram (Fig 1F). As shown in our previous reports, the clustering objectively categorizes ion conduction events into several different mechanisms [16,17].

## Results and Discussion

A part of the trajectory that is reported in our previous studies [16,17] is distributed as a tutorial of IBiSA_tools. This simulation system consists of the pore domain of the tetrameric K^+^ ion channel Kv1.2 (PDB ID 2r9r), a POPE lipid bilayer, and a 600 mM KCl solution. For the potential calculations, CHARMM27 force field [22] with CMAP [23], TIP3P water model [24], and the particle mesh Ewald method [25] were used. The simulation was performed using GROMACS, version 4.5.3 [26]. An outer electric field was applied along the Z-axis, which is perpendicular to the membrane plane, in order to imitate the membrane voltage of +920 mV [27]. The sample trajectory records 50 ns time course of the system (note that the coordinates of water molecules and Cl^−^ ions are not included) with 10 ps intervals between snapshots. This section presents the results of analyses by means of IBiSA_tools for this trajectory.

**Fig 2:**
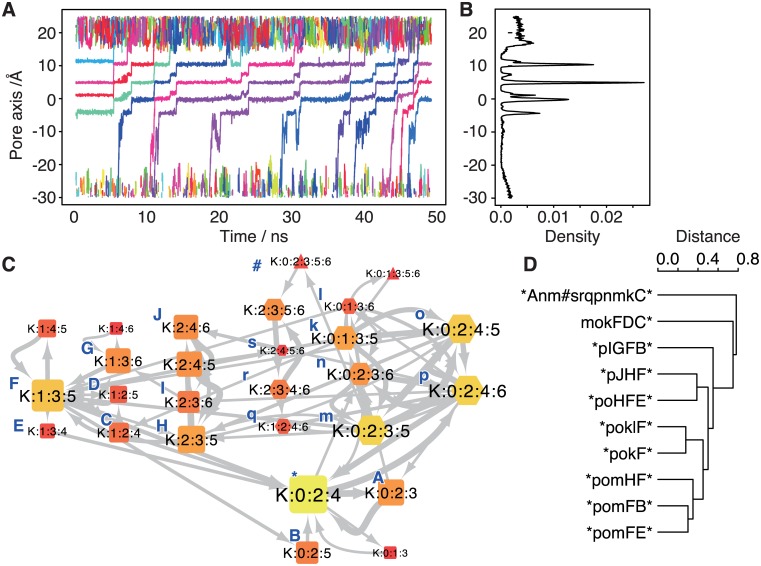
Results of the ion-binding state analysis by means of IBiSA_tools. (A) The trajectory of ions along the pore axis. The horizontal and vertical axes denote time and the pore axis coordinate. A plot in each color corresponds to a trajectory of each ion. (B) Density of the observed frequency of ions along the pore axis. (C) The graph of ion-binding states. Nodes mean the ion-binding states. The color of each node denotes stability of the state (brighter means more stable). Characters in blue beside nodes are single-character representation of the ion-binding state, corresponding to the sequence in panel D. Gray arrows mean the observed transitions between states, whose width indicates the frequency of transition. (D) A classification of ion conduction events. Sequences were defined as a cyclic path in the graph (also see S1 Fig). The dendrogram shows the result of hierarchical clustering based on the sequence alignment.

The pore axis was defined on the basis of the backbone oxygen atoms of Thr374 and those of Tyr377, which are the bottom and top of the selectivity filter, respectively. Motions of ions along this pore axis are presented in Fig 2A. During the 50 ns time course, 11 K^+^ ions permeated the pore. The plot shows that three or four ions were always retained in the pore and were temporally trapped at certain positions. An incoming ion from the intracellular fluid sometimes pushed the bound ions, and the outermost ion was released to the extracellular fluid. Although it was a rare event, an ion can return to the pore from the extracellular fluid (cyan in Fig 2A, ~22 ns). The density distribution of ions along the pore axis presented a series of discretized ion-binding sites as peaks (Fig 2B). For the following analyses, we used the definition of boundaries of ion-binding sites from other reports [16,17]. The boundaries (and the names of ion-binding sites) were 12.93, (S0), 9.32, (S1), 6.25, (S2), 3.00, (S3), 0.44, (S4), -2.21, (S5), -6.08, and (S6) -20 Å. S6 corresponds to the central cavity of the channel, which is a wide solvated region below the selectivity filter. Using this definition of the ion-binding sites, the ion-binding state of each snapshot was analyzed. The network diagram summarizes ion-binding states and transitions between them (Fig 2C). A variety of ion-binding states were observed, and state K:0:2:4 was the most stable. If we start with the most stable state, attaching an ion changes the state to a four-ion state (hexagonal nodes), and successive detachment of the outermost ion changes the state to a three-ion state (rounded square nodes on the left side of Fig 2C). Then, the system returns to the most stable state with the movements of ions in the pore. This kind of cycle represented the majority of ion conduction events observed in this trajectory. All 10 observed cyclic paths and their classification are depicted in Fig 2D. The correspondence between each character in the sequences and each state is shown in the network diagram (blue characters near nodes); the upper- and lowercase letters indicate three- and four-ion states, respectively; and symbols * and # are the most stable state and five-ion state, respectively. Eight paths started with the transition to “p” (K:0:2:4:6), meaning arrival of an ion from the intracellular fluid. Six of these paths next transitioned to “o” (K:0:2:4:5) with advancement of the new ion through the pore. In the other two paths, the outermost ion was readily pushed out and changed the system to the three-ion states (“I” and “J”). As an example, pathway *pomFE* is illustrated in S1 Fig.

There were two exceptions, *i*.*e*., paths that did not start with the transition from * to p. One path, mikFDC*, is the pathway including the first frame of the trajectory (the “m” state). The other path, *Anm#srqpnmkC*, included conduction events of two ions. After an ion was attached from the intracellular side (from A to n), the other ion was successively attached (from m to #). After that, the outermost ion was immediately detached (# to s) followed by advancement of ions through the pore and detachment of the second ion (from k to C). The dendrogram of ion conduction events helps to scrutinize the ion conduction mechanisms observed in the MD trajectory.

## Conclusions

We developed a toolkit for analyzing the detailed mechanisms of events of ion conduction through an ion channel on the basis of our original analytical method for MD trajectories. This toolkit may help to analyze complex behaviors of many ions in an ion channel. The MD trajectory of Kv1.2 channel as a tutorial demonstrates the whole process of analysis with IBiSA_tools, providing a variety of plots and statistics. In particular, the ion-binding state graph presents an overview of ion-binding states of an ion channel as an energy landscape. The dendrogram shows objective classification of ion conduction events observed in MD simulations. In our previous works, we have extensively used the above method and demonstrated that a K^+^ channel conducts K^+^ ions in two ways: the knock-on mechanism and association/dissociation mechanism [16,17]. In addition, the results showed that the preferences of ion conduction mechanisms are determined by the balance of two conditions: the ion concentration of the solvent and the membrane voltage.

In the current framework, targets of the ion-binding state analysis with IBiSA_tools have to meet the following conditions: (i) The pore axis can be defined as a straight line in 3D Cartesian space. (ii) A series of binding sites are aligned on the pore axis. (iii) Every pair of successive ion-binding sites can be discriminated by a certain threshold value (*—site-boundary* keyword in the configuration file). As described above, K^+^ channels satisfy these conditions, and this class of ion channels is the main target of IBiSA_tools. Although the scope of this analytical method is limited to biophysical phenomena related to such ion channels, elucidation of this kind of phenomena is a serious challenge in life sciences. Ion channels, which originate from the most primitive organisms, diverged to 235 genes in humans, and 78 of them are K^+^ channel genes [1]. Diseases caused by dysfunction of ion channels, which are called channelopathies, have been studied extensively. Our easy-to-use analytical toolkit can facilitate research in this important field.

Software IBiSA_tools is distributed under an open source license at the following URL: http://www.ritsumei.ac.jp/~ktkshr/ibisa_tools/

## Supporting Information

S1 Fig A schematic diagram of an ion conduction event, *pomFE*. (A) The cyclic path mapped onto the ion-binding state graph (Fig 2C). The colored arrows (red, orange, green, cyan, blue, and purple) indicate transitions of the ion-binding state. (B) Another representation of the same ion conduction event. Boxes and balls indicate ion-binding sites and K^+^ ions, respectively. The numbers from 0 to 6 are IDs of each ion-binding site.
(EPS)
